# The association of metabolic syndrome components and chronic kidney disease in patients with hypertension

**DOI:** 10.1186/s12944-019-1121-5

**Published:** 2019-12-27

**Authors:** Kun Xie, Liwen Bao, Xiaofei Jiang, Zi Ye, Jianping Bing, Yugang Dong, Danchen Gao, Xiaoping Ji, Tingbo Jiang, Jiehua Li, Yan Li, Suxin Luo, Wei Mao, Daoquan Peng, Peng Qu, Shangming Song, Hui Wang, Zhaohui Wang, Biao Xu, Xinhua Yin, Zaixin Yu, Xinjun Zhang, Zixin Zhang, Zhiming Zhu, Xiufang Gao, Yong Li

**Affiliations:** 1 Cardiology Department, Huashan Hospital, Fudan University, No. 12 Mid Wulumuqi Road, Shanghai, 200040 China; 2 Department of cardiovascular medicine Mayo Clinic, Rochester, USA; 3 Nanfang Hospital, Guangzhou, China; 4 The First Affiliated Hospital of Sun Yat-sen University, Guangzhou, China; 5 The First Affiliated Hospital of Zhejiang University, Hangzhou, China; 6 Qilu Hospital of Shandong University, Jinan, China; 7 The First Affiliated Hospital of Soochow University, Suzhou, China; 8 The First Affiliated Hospital of Anhui Medical University, Hefei, China; 9 Rui Jin Hospital Shanghai Jiao Tong University School of Medical, Shanghai, China; 10 The First Affiliated Hospital of Chongqing Medical University, Chongqing, China; 11 Zhejiang Provincial Hospital of TCM, Zhejiang, China; 12 The Second Xiangya Hospital of Central South University, Furong, China; 13 The second hospital of Dalian Medical University, Zunyi, China; 14 Shandong Provincial Hospital, Jinan, China; 15 Jiangsu Province Hospital, Nanjing, China; 16 Wuhan Union Hospital of, Wuhan, China; 17 Nanjing Drum Tower Hospital, The Affiliated Hospital of Nanjing University Medical School, Nanjing, China; 18 First Affiliated Hospital of Harbin Medical University, Harbin, China; 19 Xiangya Hospital, Central South University, Changsha, China; 20 West China Hospital, Sichuan University, Lesha, China; 21 The First Affiliated Hospital of China Medical University, Shenyang, China; 22 Grand ping hospital, third military medical university, Chongqing, China

**Keywords:** Hypertension, Metabolic syndrome, Triglyceride, Chronic kidney disease

## Abstract

**Background:**

Hypertension is a highly prevalent disease and the leading cause of chronic kidney disease (CKD). Metabolic syndrome could also be the risk factor for CKD. We sought to study the association between metabolic syndrome components and the prevalence of CKD in patients with hypertension.

**Methods:**

We carried out a multi-center cross-sectional study from Apr. 2017- Apr. 2018 in 15 cities in China.

**Results:**

A total of 2484 patients with hypertension were enrolled. Among them, 56% were male and the average age was 65.12 ± 12.71 years. The systolic BP/diastolic BP was 142 ± 18/83 ± 12 mmHg. Metabolic syndrome components turned out to be highly prevalent in patients with hypertension, ranging from 40 to 58%. The prevalence of chronic kidney disease reached 22.0%. Multi-variate logistic analysis revealed that elevated triglyceride (TG) (OR = 1.81, 95% CI 1.28–2.57, *p* < 0.01), elevated fasting blood glucose (FBG) (OR = 1.43, 95% CI 1.00–2.07, *p* = 0.05) and hypertension grades (OR = 1.20, 95% CI 1.00–1.44, p = 0.05) were associated with the prevalence of CKD. In sub-group analysis, elevated TG remained strongly associated with CKD in both diabetes (OR = 2.10, 95%CI 1.22–3.61, *p* < 0.01) and non-diabetes (OR = 1.53, 95% CI 1.09–2.16, *p* = 0.01). In sub-group analysis of hypertension grades, there was also a graded trend between elevated TG and CKD from controlled blood pressure (BP) to hypertension grade 2 (OR = 1.81, 95%CI 1.06–3.11, *p* = 0.03; OR = 1.85, 95%CI 1.00–3.43, *p* = 0.05; OR = 2.81, 95% CI 1.09–7.28, p = 0.03, respectively).

**Conclusion:**

Elevated TG, elevated FBG and hypertension grades were significantly associated with the prevalence of CKD in patients with hypertension. Particularly, elevated TG was strongly associated with CKD, independent of diabetes and hypertension grades.

## Introduction

Hypertension is highly prevalent worldwide. It has been reported that 31.1% (95%CI 30.0–32.2%) of the world’s adults have hypertension. However, the awareness, treatment and control rate are far from satisfying. Data from different countries showed that the awareness of hypertension ranged from 37.9–67%, treatment rate ranged from 29.0–55.6% and the control rate ranged only from 7.7–28.4% [1]. Hypertension is one of the leading causes of CKD. In the US, 28.4% of the end stage CKD were caused by hypertension from 1980 to 2010. The increasing number of CKD is becoming the common public health problem which demands large quantity of health resources and leads to poor quality of life. It’s also well known that CKD is associated with increased cardiovascular and all-cause mortality. Additionally, several studies have suggested that not only hypertension, but also metabolic syndrome should be considered as the risk factor for CKD. Although the awareness of metabolic syndrome is improving, there are still 50% of the population older than 60 years being diagnosed to have metabolic syndrome [2]. Some data have demonstrated that metabolic syndrome was associated with either the prevalence or the incidence of CKD [3, 4], but there are still discrepancies regarding the effect of each metabolic syndrome components on the development of CKD.Moreover, to our knowledge, it is still unclear how much effect the other metabolic syndrome components, besides hypertension, will have on the development of CKD. To fill this gap, we carried out a cross-sectional study to investigate whether there are independent association between metabolic syndrome components and CKD in patients with hypertension.

## Methods

We carried out a multi-center cross-sectional study from Apr. 2017-Apr. 2018 in the main 15 cities in China. Participants were enrolled from hypertension clinic using convenience sampling. Inclusion criteria were patients with primary hypertension, aged over 18 years, who could provide informed consents. Patients were excluded if they had secondary hypertension, genetic renal diseases, active cancer or recognition disorder. Each patient completed a questionnaire collecting information on demographic characteristics, medical history, lifestyle features, current medical treatment, physical examination and lab tests.

According to American Heart Association, the diagnosis of metabolic syndrome includes any 3 of the 5 criteria: 1) Elevated waist circumference > =90 cm in men and > =80 cm in women (for Asian); 2) Elevated triglycerides (TG) > =1.7 mmol/L; 3) Reduced HDL-C < 1.03 mmol/L in men or < 1.3 mmol/L in women; 4) Elevated blood pressure > =130/85 mmHg or on antihypertensive drug treatment with a history of hypertension; 5) Elevated fasting blood glucose (FBG) > =5.6 mmol/L or on drug treatment for elevated glucose [5].

As is listed above, in our study, elevated waist circumference was defined as waist circumference > =90 cm in men and > =80 cm in women; elevated TG was defined as TG > =1.7 mmol/L; reduced HDL-C was defined as HDL-C < 1.03 mmol/L in men or < 1.3 mmol/L in women; elevated FBG was defined as FBG > =5.6 mmol/L or on drug treatment for elevated glucose.

Hypertension grades was defined according to 2013 ESC/ESH Guidelines for the management of arterial hypertension [6] . Controlled BP was defined as systolic blood pressure (SBP) < 140 mmHg and/or diastolic blood pressure (DBP) < 90 mmHg; Grade 1 was defined as SBP 140–159 mmHg and/or DBP 90–99 mmHg; Grade 2 was defined as SBP 160–179 mmHg and/or DBP 100–109 mmHg; Grade 3 was defined as SBP > =180 mmHg and/or DBP > =110 mmHg.

CKD was defined as estimated glomerular filtration rate (eGFR) < 60 ml/min/1.73m^2^, while eGFR was calculated using Modification of Diet in Renal Disease Study equation [7].

Baseline characteristics were analyzed and compared in patients with and without CKD. Either two-side Student’s t-test or Wilcoxon rank sum test were used for continuous variables. Chi-square test was used for categorical variables. A forward stepwise method was used to reach the logistic regression model. Odds Ratios (OR) and 95% confidence intervals from logistic regression were used to estimate the association between metabolic syndrome components and CKD. Sub-group analysis was conducted according to concomitant diabetes and hypertension grades. P < =0.05 was considered statistically significant. STATA 13.0 was used for statistical analysis.

## Results

In the 2484 patients with hypertension, the average age was 65.12 ± 12.71 years. Male accounted for 55.9% of the total participants. The average BP was 142.30 ± 17.68 / 83.01 ± 11.93 mmHg. The distribution of hypertension grade was 41.6, 36.9, 14.9 and 6.7% separately. Totally 90.7% of them were taking antihypertensive therapy.

Metabolic syndrome turned out to be highly prevalent. Patients with elevated TG, reduced HDL-C, elevated FBG and elevated waist circumference were 46.9, 40.1, 58.3 and 47.5% separately. Besides, 24.7% of them had diabetes and 24.2% of them were taking antidiabetic therapy. Additionally, 60.3% were using statins and 0.8% were using fibrates.

The prevalence of chronic kidney disease was also high, reaching 22.0% of the total participants (Table 1).

**Table 1:** The characteristics of the total participants and patients with or without CKD

	Total (*N* = 2484)	CKD (−) (*N* = 1937)	CKD (+) (*N* = 547)	*p*
Age (year)	65.12 ± 12.71	64.28 ± 12.53	68.09 ± 12.94	< 0.01
Male (%)	55.9%	57.9%	49.0%	< 0.01
Systolic BP (mmHg)	142.30 ± 17.68	142.15 ± 17.64	142.81 ± 17.81	0.44
Diastolic BP (mmHg)	83.01 ± 11.93	82.92 ± 12.09	83.35 ± 11.34	0.45
Hypertension grades (%)				0.16
Controlled	41.6%	42.8%	37.5%	
1	36.9%	36.3%	39.0%	
2	14.9%	14.4%	16.5%	
3	6.7%	6.6%	7.0%	
Antihypertensive therapy (%)	90.7%	90.7%	90.7%	0.99
TG (mmol/L)	1.62(1.14, 2.3)	1.60(1.11–2.26)	1.77(1.28–2.42)	< 0.01
Elevated TG (%)	46.9%	45.2%	54.1%	0.01
HDL-C (mmol/L)	1.21(1.01–1.48)	1.21(1.02–1.46)	1.2(0.99–1.53)	0.90
Reduced HDL-C (%)	40.1%	39.5%	42.5%	0.35
Statin (%)	60.3%	58.2%	67.1%	< 0.01
FBG (mmol/L)	6.38 ± 2.14	6.31 ± 2.01	6.72 ± 2.62	0.01
Elevated FBG (%)	58.3%	56.6%	66.1%	0.01
Diabetes (%)	24.7%	23.7%	28.3%	0.03
Antidiabetic therapy (%)	24.2%	22.8%	29.2%	< 0.01
Waist circumference (cm)	84.18 ± 14.75	83.71 ± 13.89	85.86 ± 17.35	< 0.01
Elevated waist circumference (%)	47.5%	45.7%	54.0%	< 0.01
eGFR (ml/min/1.73 m2)	79.24 ± 29.18	91.16 ± 18.87	37.03 ± 17.91	< 0.01
CKD	22.0%	–	–	–

*BP*: Blood pressure; *CKD*: Chronic kidney disease; *eGFR*: estimated glomerular filtration rate; *FBG*: Fasting blood glucose; *HDL-C*: High density lipoprotein-C; *TG*: Triglycerides;

We compared the characteristics of patients with and without CKD. The average age was 64.3 ± 12.5 years in non-CKD group and 68.09 ± 12.94 years in CKD group (*p* < 0.01). Among them, 57.9% were male in non-CKD group and 49.0% were male in CKD group (p < 0.01). No difference was found in the distribution of hypertension grades (*p* = 0.16) and antihypertensive therapy (*p* = 0.99) between the two groups. The ratio of elevated TG, reduced HDL and elevated FBG were 45.2,39.5 and 56.6% in non-CKD group compared to 54.1% (*p* = 0.01), 42.5% (*p* = 0.35) and 66.1% (*p* = 0.01) in CKD group. The elevated waist circumference was 45.7% in the non-CKD group compared to 54.0% in the CKD group (*p* < 0.01). The prevalence of DM was 23.7% in non-CKD group compared to 28.3% in CKD group (*p* = 0.03). Totally 22.8% of the patients in non-CKD group were taking antidiabetic therapy compared to 29.2% in CKD group (*p* < 0.01). Additionally, 58.2% patients were using statins in non-CKD group compared to 67.1% in CKD group (*p* < 0.01) (Table 1).

The association between metabolic syndrome components and prevalence of CKD were explored (Table 2). Unadjusted analysis showed that CKD was associated with elevated TG, elevated FBG and elevated waist circumference, but not with reduced HDL-C or hypertension grades. After adjusting for age and gender, it showed that except reduced HDL-C, all the other elements including hypertension grades, elevated TG, elevated FBG and elevated waist circumference were strongly associated with CKD. While after further adjustment for age, gender, antihypertensive therapy and statin therapy, the association remained in hypertension grades (OR = 1.20, 95% CI 1.00–1.44, *p* = 0.05), elevated TG (OR = 1.81, 95% CI 1.28–2.57, *p* < 0.01) and elevated FBG (OR = 1.43, 95% CI 1.00–2.07, *p* = 0.05).

**Table 2:** The Odds ratio of metabolic syndrome components for the prevalence of CKD

	Model 1		Model 2		Model 3	
	OR (95% CI)	*p*	OR (95% CI)	*p*	OR (95% CI)	*p*
Hypertension grades	1.10 (1.00–1.22)	0.06	1.15 (1.04–1.28)	0.01	1.20 (1.00–1.44)	0.05
Elevated TG	1.43 (1.11–1.83)	0.01	1.63 (1.26–2.11)	< 0.01	1.81 (1.28–2.57)	0.01
Reduced HDL-C	1.13 (0.88–1.46)	0.35	1.07 (0.82–1.39)	0.64	0.79 (0.55–1.14)	0.21
Elevated FBG	1.85 (1.37–2.49)	< 0.01	1.83 (1.36–2.47)	< 0.01	1.43 (1.00–2.07)	0.05
Elevated waist circumference	1.40 (1.15–1.69)	< 0.01	1.31 (1.07–1.59)	0.01	1.28 (0.90–1.82)	0.17

Model 1: unadjusted OR; Model 2: OR adjusted for age and gender; Model 3: OR adjusted for age, gender, antihypertensive therapy, statin therapy. *FBG*: Fasting blood glucose; *HDL-C*: High density lipoprotein-C; *TG*: Triglycerides

As diabetes plays a key role in the development of CKD, we also carried out sub-group analysis investigating whether metabolic syndrome components would still be associated with CKD in patients either with or without diabetes.

The association between hypertension grades and CKD remained strongly significant in both unadjusted and multivariate adjusted models in diabetes patients (OR = 1.37, 95%CI 1.04–1.82, *p* = 0.03). However, such association was not found in non-diabetes patients (OR = 0.791, 95%CI 0.85–1.24, *p* = 0.79).

Elevated TG was always independently associated with CKD, not only in patients with diabetes (OR = 2.10, 95%CI 1.22–3.61, *p* = 0.01), but also in patients without diabetes (OR = 1.53, 95% CI 1.09–2.16, *p* = 0.01).

Reduced HDL-C was not associated with CKD in patients either with diabetes (OR = 0.66, 95%CI 0.39–1.13, *p* = 0.13) or without diabetes (OR = 1.18, 95%CI 0.83–1.68, *p* = 0.37).

Similarly, elevated waist circumference was not associated with CKD in patients either with diabetes (OR = 1.28, 95% CI 0.74–2.20, p = 0.37) or without diabetes (OR = 1.00, 95%CI 0.70–1.40, *p* = 0.98) (Table 3).

**Table 3:** The Odds ratio for the prevalence of CKD in patients with or without T2DM

	Model 1		Model 2		Model 3	
	OR (95% CI)	*p*	OR (95% CI)	*p*	OR (95% CI)	*p*
Diabetes (+)						
Hypertension grades	1.37 (1.12–1.68)	< 0.01	1.38 (1.13–1.69)	< 0.01	1.37 (1.04–1.82)	0.03
Elevated TG	1.79 (1.12–2.87)	0.02	1.95 (1.21–3.15)	0.01	2.10 (1.22–3.61)	0.01
Reduced HDL-C	0.81 (0.51–1.29)	0.37	0.79 (0.49–1.27)	0.33	0.66 (0.39–1.13)	0.13
Elevated waist circumference	1.43 (0.97–2.09)	0.07	1.47 (1.00–2.17)	0.05	1.28 (0.74–2.20)	0.37
Diabetes (−)						
Hypertension grades	1.03 (0.91–1.17)	0.64	1.08 (0.95–1.23)	0.23	1.03 (0.85–1.24)	0.79
Elevated TG	1.35 (0.99–1.82)	0.06	1.54 (1.12–2.12)	0.01	1.53 (1.09–2.16)	0.01
Reduced HDL-C	1.29 (0.94–1.76)	0.12	1.23 (0.89–1.71)	0.21	1.18 (0.83–1.68)	0.37
Elevated waist circumference	1.30 (1.03–1.63)	0.03	1.18 (0.93–1.49)	0.17	1.00 (0.70–1.40)	0.98

Model 1: unadjusted OR; Model 2: OR adjusted for age and gender; Model 3: OR adjusted for age, gender, antihypertensive therapy and statin therapy. *DM*: Diabetes mellitus; *FBG*: Fasting blood glucose; *HDL-C*: High density lipoprotein-C; *TG*: Triglycerides

As elevated TG was always independently associated with CKD, we further explored the association between elevated TG and CKD in subgroups according to hypertension grades.

We found that the association between elevated TG and CKD tend to increase from BP controlled (OR = 1.81, 95%CI 1.06–3.11, *p* = 0.03), grade 1 (OR = 1.85, 95%CI 1.00–3.43, *p* = 0.05) to grade 2 (OR = 2.81, 95% CI 1.09–7.28, p = 0.03). While only in hypertension grade 3, elevated TG was not associated with CKD (OR = 1.88, 95%CI 0.44–7.97, *p* = 0.39) (Table 4).

**Table 4:** The association between elevated TG and the prevalence of CKD in subgroup analysis of hypertension grades

Hypertension grades	Adjusted OR	95% CI	*p*
Controlled	1.81	1.06–3.11	0.03
1	1.85	1.00–3.43	0.05
2	2.81	1.09–7.28	0.03
3	1.88	0.44–7.97	0.39

The multivariate logistic analysis was adjusted for age, gender, antihypertensive therapy and statin therapy

## Discussion

In this study we found that metabolic syndrome components, including elevated TG, elevated FBG and hypertension grades were associated with CKD in patients with hypertension. The subgroup analysis showed that elevated TG, particularly, was associated with CKD, independent of diabetes and hypertension grades.

The association between metabolic syndrome and CKD has been evaluated by a few studies but the results were inconclusive. Back in 2004, Chen et al. reported that in a subsample of 7832 participants in the third National Health and Nutrition Examination Survey (NHANES III) in the US, participants with metabolic syndrome had increased odds for chronic kidney disease compared to those without metabolic syndrome [3]. In another observational prospective cohort study with 10,096 nondiabetic participants, metabolic syndrome was independently associated with the incidence of CKD during a 9-year follow-up^4^. Data from a Chinese cross-sectional study with 2310 participants 40 years or older showed that metabolic syndrome was independently associated with CKD, especially the components of elevated TG, elevated BP and elevated FBG [8], which was quite consist with the result of our study. More recently, in a cohort study in Kyushu and Okinawa with 1824 participants during a five-year follow-up, hypertriglyceridemia and carotid intima-media thickness (IMT) were found to be independently associated with the development of CKD [9]. In a retrospective registry of 3748 patients with type 2 DM, Aaman et all found that a higher level of plasma triglyceride was associated with CKD in Thailand [10]. In the year of 2018, A meta-analysis which included 692,909 CKD and 11,040,527 participants illustrated that metabolic syndrome and all of its 5 components were significantly associated with CKD. Furthermore, there was a graded trend between the increasing number of metabolic syndrome components and the risk of CKD [11].

Except that hypertension and diabetes have long been documented to be the major risks for the progression of CKD, elevated TG was found in our study to be associated with CKD, particularly independent of diabetes and hypertension grades. The mechanism of how the lipid level affect the injury of renal function was still incompletely understood. There are several mechanic pathways involved, including atherosclerosis, oxidative stress, chronic inflammation, hemodynamic change and endothelial dysfunction. For patients with dyslipidemia, specifically high TG, direct stimulation of glomerulosclerosis may be the key mechanism [2]. In fact, previous studies reported the association between various types of dyslipidemia and CKD besides elevated TG, including TC, non-HDL-C, HDL-C, TG/HDL-C or TC/HDL-C [12–14], which were all considered to be atherogenic. Further studies are needed to explain the mechanism of the impact of elevated TG on the loss of kidney function.

TG has been strongly debated to be a causal factor driving atherosclerosis [15]. There are still residual risks which may be attribute to the elevated TG once LDL-C target has been achieved. For patients with hypertension, the adding of statin to antihypertensive treatments has been well established to reduce LDL-C. However, the awareness of TG management hasn’t been satisfying. But now, at least, TG should be included in the risk management of patients with hypertension.

The main weakness of our study was the relatively small sample size with convenience sampling, by which the possibility of bias might exist. In addition, since it was a cross-sectional study rather than a causal design, it was difficult to tell the causal relationship between metabolic syndrome components and CKD. We could only conclude that there was a strong independent association between them.

However, the strength of the study was that it was a multicenter cross-sectional study collecting data from all over the country, which made it possible for the conclusion to be generalized to all the patients with hypertension in China. Second, to our knowledge, this was the first study to evaluate the risk of CKD in patients with hypertension and other metabolic syndrome components. The importance of metabolic syndrome management in patients with hypertension should be further emphasized. Last, more attention should be focused on TG during risk management in order to prevent CKD and further research into the mechanism behind is needed.

## Conclusion

In our cross-sectional study of patients with hypertension, elevated TG, elevated FBG and hypertension grades were independently associated with the prevalence of CKD. Particularly, elevated TG was strongly associated with CKD, independent of diabetes and hypertension grades. Thus, among all the metabolic syndrome components, TG should also be concerned during the risk management of hypertension.

## Supplementary information

**Additional file 1.** The ethics approval.

**Additional file 2.** The patients' consent.

## Data Availability

The datasets during and/or analyzed during the current study will be available from the corresponding author on reasonable request.

## Abbreviations

CKD: Chronic Kidney Disease
DBP: Diastolic Blood Pressure
eGFR: Estimated Glomerular Filtration Rate
FBG: Fasting Blood Glucose
HDL-C: High Density Lipoprotein-Cholesterol
IMT: Intima-Media Thickness
LDL-C: Low Density Lipoprotein-Cholesterol
OR: Odds Ratio
SBP: Systolic Blood Pressure
TC: Total Cholesterol
TG: Triglycerides
