# Supplementary figures and images for: The association of metabolic syndrome components and chronic kidney disease in patients with hypertension
Source: Lipids Health Dis. 2019 Dec 27;18:229. doi: 10.1186/s12944-019-1121-5 (PMC6935087; doi:10.1186/s12944-019-1121-5)

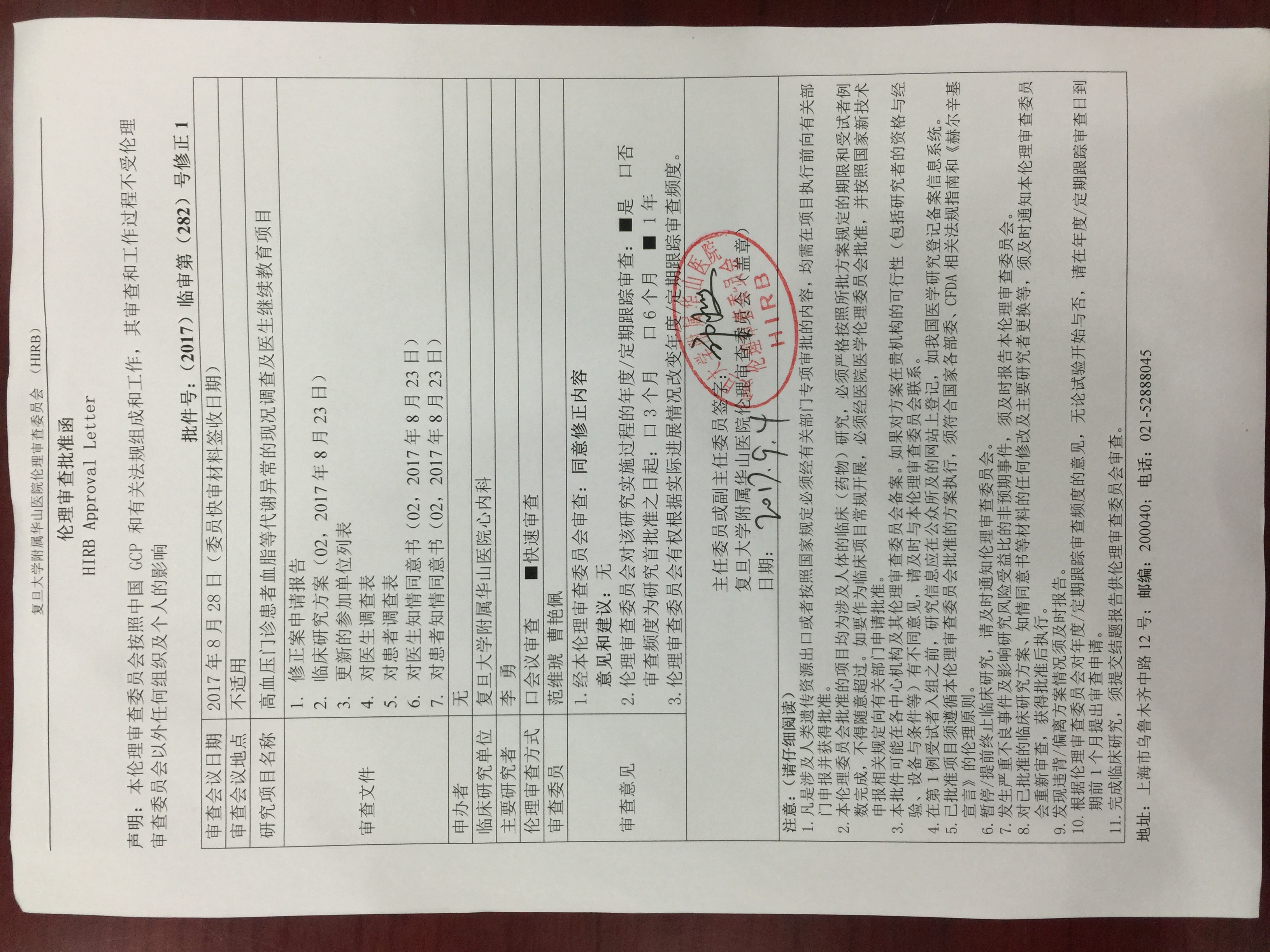

Supplement: Supplementary file 1 — Additional file 1. The ethics approval. [file 12944_2019_1121_MOESM1_ESM.jpg]
