# Supplementary material for: The association of metabolic syndrome components and chronic kidney disease in patients with hypertension
Source: Lipids Health Dis. 2019 Dec 27;18:229. doi: 10.1186/s12944-019-1121-5 (PMC6935087; doi:10.1186/s12944-019-1121-5)
Supplement: Supplementary file 2 — Additional file 2. The patients' consent. [file 12944_2019_1121_MOESM2_ESM.pdf]

受试者须知

（患者）

方案名称：高血压门诊患者血脂等代谢异常的现况调查及医生继续教育项目

Survey on BP, Lipids and cardiovascular risks in the outpatient hypertensives and Continuing Medical Education Program（UPPDATE）

方案编号：KY2017-282

知情同意书版本号：02，2017 年 8 月 23 日

研究机构：复旦大学附属华山医院

主要研究者：李勇

您将被邀请参加一项临床研究。本须知提供给您一些信息以帮助您决定是否参加此项临床研究。请您仔细阅读，如有任何疑问请向负责该项研究的研究者提出。

您参加本项研究是自愿的。本次研究已通过本研究机构伦理审查委员会审查。

研究目的：2015 年心血管病报告显示我国高血压患者达 2.7 亿，血脂异常患者超过 1 亿，高血压治疗率约为 1/3，而血脂异常治疗率不足 1/3。高血压和血脂异常常伴发。有研究显示我国门诊高血压患者 81.1%合并血脂异常。高血压患者降胆固醇治疗仍然具有巨大的改进空间。

这项调查研究的目的在于帮助您获得更规范的高血压治疗方案。通过了解您的心血管危险因素（包括血脂水平等）、降压药物使用情况及血压控制情况、合并代谢异常的情况及其控制情况，您的医生将全面地了解您的病情和治疗情况，有利于医生发现您治疗中的不足，帮助医生制定针对您的个体化血压控制和代谢异常的治疗方案。

研究过程：本研究主要调查高血压患者的代谢异常情况，以及血压和血脂等的治疗情况。如果您同意参与这项研究，我们将对您进行编号，建立病历档案。在研究过程中我们需要收集您的疾病信息，包括您的目前状况、既往病史、既往检查化验的结果和治疗情况。

风险与不适：对于您来说，所有的信息将是保密的。这只是观察性的研究，即在日常治疗中收集数据。您仍由您的医生按照常规进行治疗。

受益：通过了解您的心血管危险因素（包括血脂水平等）、降压药物使用情况及血压控制情况、合并代谢异常的情况及其控制情况，您的医生将全面地了解您的病情和治疗情况，

有利于医生发现您治疗中的不足，帮助医生制定针对您的个体化血压控制和代谢异常的治疗方案。

作为研究受试者，您有以下职责：提供有关自身病史和当前身体状况的真实情况；尽可能地提供近一年内的检查化验报告；尽可能全面准确地告诉医生您目前的用药情况。告诉研究医生自己在最近是否曾参与其他研究，或目前正参与其他研究。

隐私问题：如果您决定参加本项研究，您参加研究及在研究中的个人资料均属保密。可以识别您身份的信息将不会透露给研究小组以外的成员，除非获得您的许可。所有的研究成员和研究申办方都被要求对您的身份保密。您的档案将保存在有锁的档案柜中，仅供研究人员查阅。为确保研究按照规定进行，必要时，政府管理部门或伦理审查委员会的成员按规定可以在研究单位查阅您的个人资料。这项研究结果发表时，将不会披露您个人的任何资料。

您可以选择不参加本项研究，或者在任何时候通知研究者要求退出研究，您的数据将不纳入研究结果。

如果您没有遵守研究计划，或者有任何其它原因，研究医师可以终止您继续参与本项研究。

如果您有与本研究有关的问题，或有关于本项研究参加者权益方面的问题您可以通过_021-52887064_____与 高秀芳___联系。

如果您对参与本研究的权益和健康有任何问题或诉求，您可以联系本机构伦理委员会，联系电话：52888045；联系人：吴翠云。

知情同意书签字页

我已经阅读了本知情同意书。

我有机会提问而且所有问题均已得到解答。

我理解参加本项研究是自愿的。

我可以选择不参加本项研究，或者在任何时候通知研究者后退出而不会遭到歧视或报复，我的权益不会因此而受到影响。

如果我没有遵守研究计划，或者有任何其它原因，研究医师可以终止我继续参与本项研究。

我将收到一份签过字的"知情同意书"副本。

受试者姓名：________________________

受试者签名：_________________________

日期：_______年________月________日

我已准确地将这份文件告知受试者，他/她准确地阅读了这份知情同意书，并有机会提出问题。

研究者姓名：________________________

研究者签名：_________________________

日期：_______年________月________日

（*注：如果受试者不识字时尚需见证人签名，如果受试者无行为能力时则需代理人签名*）
